# Effect of Genistein on vasculogenic mimicry formation by human uveal melanoma cells

**DOI:** 10.1186/1756-9966-28-124

**Published:** 2009-09-07

**Authors:** Rihong Cong, Qingmin Sun, Li Yang, Haijuan Gu, Ying Zeng, Bin Wang

**Affiliations:** 1 Department of Pharmacology, Nanjing Medical University, Nanjing 210029, PR China; 2 Department of General Surgery, First Affiliated Hospital of Nanjing Medical University, Nanjing 210029, PR China

## Abstract

**Background:**

Vasculogenic mimicry (VM) was increasingly recognized as a form of aggressive melanoma acquiring blood supply. Genistein had attracted much attention as a potential anticancer agent. Therefore, we examined the effect of Genistein on VM in human uveal melanoma cells.

**Methods:**

VM structure was detected by periodic acid-Schiff (PAS) staining for uveal melanoma C918 cells cultured on the three-dimensional type I collagen gels after exposed to Genistein. We used reverse transcription polymerase chain reaction (RT-PCR) and Western Blot analysis to examine the effect of Genistein on vascular endothelial cadherin (VE-cadherin) mRNA and protein expression. The nude mice models of human uveal melanoma C918 cells were established to assess the number of VM using immunohistochemical and PAS double-staining.

**Results:**

Genistein inhibited the survival of C918 cells in vitro. The ectopic model study showed that VM in tumor tissue sections were significantly reduced by Genistein in vivo. In vitro, the VM structure was found in control, 25 and 50 μM Genistein-treatment groups but not in 100 and 200 μM. RT-PCR and Western Blot showed that 100 and 200 μM concentration of Genistein could significantly decrease VE-cadherin mRNA and protein expression of C918 cells compared with control (P < 0.05). However, the 25 and 50 μM Genistein slightly decreased the VE-cadherin level in vitro (P > 0.05).

**Conclusion:**

Genistein inhibits VM formation of uveal melanoma cells in vivo and in vitro. One possible underlying molecular mechanism by which Genistein could inhibit VM formation of uveal melanoma is related to down-regulation of VE-cadherin.

## Background

Malignant tumor growth, progression, and metastasis depend on adequate blood supply [1]. Much attention has been focused on angiogenesis which is known as the sprouting of new vessels from existing microvessels. The traditional anticancer treatment is targeting the vascular and endothelial cells [2,3]. In 1999, Maniotis and co-workers introduced the concept of vasculogenic mimicry (VM), a new mechanism by which aggressive melanoma may acquire a blood supply [4]. VM channels are patterned networks of interconnected loops of periodic acid-Schiff (PAS)-positive extracellular matrix forming by aggressive melanoma tumor cells instead of endothelial cells. Moreover, it is correlated with poor prognosis in patients with tumors [4] and has been described in several other aggressive tumor types [5-8]. Uveal melanoma, the most common primary intra-ocular tumor in adults, has been widely concerned as the purely hematogenous [9]. Nearly 50% of uveal melanoma patients die from metastatic melanoma [10]. However, no effective therapeutic modalities are available for preventing metastases or improving the survival rate of uveal melanoma patients.

Genistein is a predominant isoflavone in soybeans and has been shown to inhibit the invasion and growth of various cancer cells including prostate, breast, lung, head and neck cancer [11-14]. The anticancer mechanism of Genistein has been illustrated to inhibit angiogenesis both in vivo and in vitro [15]. Our previous work also found that Genistein was capable to inhibit ocular neovascularization through suppression of vascular endothelial growth factor (VEGF), hypoxia inducible factor 1 (HIF 1) and basic fibroblast growth factor (bFGF) expression [16-19]. Genistein inhibit endothelial cells proliferation. Moreover, melanoma cells could imitate endothelial cells to form VM channels and expressed some endothelial-associated genes, including vascular endothelial cadherin (VE-cadherin, a calcium-dependent adhesion molecule). Therefore, this study was performed to evaluate the effect of Genistein on the VM channels formation of highly aggressive melanoma cells. In addition, it has been indicated that VE-cadherin plays a critical role in the formation of melanoma VM [20,21]. We also examined the influence of Genistein on VE-cadherin level and explored the underlying molecular mechanisms of VM.

## Materials and methods

### Drug

Genistein was purchased from Sigma (St. Louis, Missouri, USA) and dissolved in dimethylsulfoxide (DMSO) at the concentration of 200 × 10^3 ^μM. Then it was diluted with RPMI 1640 to the desired concentration. Final concentration of DMSO in cell culture medium was 0.1% (v/v). The medium containing 0.1% DMSO only served as control.

### Cell culture

The highly aggressive C918 and poorly aggressive OCM-1A human uveal melanoma cell lines were generously supplied by Prof. Elisabeth A Seftor (Children's Memorial Research Center, Chicago, IL). The cells were maintained in RPMI 1640 (Invitrogen) supplemented with 10% fetal bovine serum and 0.1% gentamicin sulfate at 37°C in an atmosphere of 5% CO_2_. After treatment with Genistein, cell proliferative activity was determined by the MTT (3-[4,5-dimethylthiazol-2-yl]-2,5 diphenyl tetrazolium bromide) assay.

### Three-dimension culture and PAS-staining

Three-dimensional type I collagen gels were produced as follows [22]: Fifty μl of type I collagen (3.02 mg/ml; BD Bioscience, Bedford, MA) were dropped onto 18-mm glass coverslips in six-well tissue culture plate. Absolute ethanol was added to each well, and the collagen was allowed to polymerize for 5 min at room temperature. After a wash with PBS, 1 × 10^6 ^C918 cells or OCM-1A cells were plated onto the three-dimensional type I collagen gels to analyze the ability of the cells to engage in VM. After 48h, the cells were fixed with 4% formaldehyde in PBS for 10 min. To identify the matrix-associated patterned networks of uveal melanoma, coverslips containing three-dimensional cultures were stained with PAS, omitting hematoxylin counterstaining [4]. Different concentrations of Genistein (0, 25, 50, 100, and 200 μM) was added to the cells to observe the effect of Genistein on VM.

### Animal model and CD34-PAS dual staining

All animal experiments were approved by the local animal ethics committee. Six week old female BALB/C nu/nu mice were purchased from Vital River Laboratory Animal Technology (Beijing, China). All experiments were performed in accordance with the official recommendations of the Chinese Community Guidelines. The xenografts were established using C918 cells [23], which were resuspended at a density of 1 × 10^7^/ml. The suspension (0.1 ml/10 g body weight) was injected subcutaneously into the nude mice. After 6 days, tumor nodules were palpable. Then the mice were randomly assigned into control and Genistein groups: control (n = 5), injected intraperitoneally with 1% DMSO/day; Genistein (n = 5), injected intraperitoneally with Genistein 75 mg/kg/day. The treatment was continued every day for 30 days. At the end, mice were sacrificed by cervical decapitation and the tumors were removed and weighed. C918 xenograft specimens were fixed in 10% neutral buffered formalin and paraffin-embedded. Paraffin-embedded specimens were cut into serial 5-μm sections. And the sections were deparaffinized, rehydrated, and subjected to immunohistochemical and PAS double-staining. The immunohistochemistry was conducted with monoclonal mouse antibodies to the endothelium marker CD34 (1:50 dilution, Beijng, Zhong Shan Goldenbridge) to identify endothelium. DAB chromogen was used for the immunohistochemistry. CD34 staining helped to distinguish the PAS-positive network of VM from endothelium-lined micro vessels. Tissues were stained with PAS to identify the matrix-associated vascular channels of uveal melanoma. Quantification of VM was performed as follow [24]: The CD34-PAS dual staining sections were viewed at × 400. The channels defined as VM were lined by PAS-positive material with red cells in the center of the channels, but not lined by CD34-positive endothelial cells. The mean VM count of ten areas was calculated as the VM density (VMD) respectively for each section. The mean VMD from 5 xenograft specimens in the Genistein and control groups were obtained as the final VMD count.

### Semiquantitative RT-PCR analysis

The mRNA expression of VE-cadherin in C918 cells was analyzed by reverse transcription polymerase chain reaction (RT-PCR). At the end of Genistein treatment, total RNA from C918 and OCM-1A cells cultured on a type I collagen three-dimensional matrix was extracted using Trizol reagent (Invitrogen) as the manufacturer's protocol. The first-strand cDNA was synthesized from 3 μg of RNA by standard reverse transcription (RT) methods, using M-MuLV reverse transcriptase (MBI Fermentas, Vilnius, Lithuania) and oligt (d) T primer according to the manufacturer's instructions. Specific primers (forward: 5'-CACCGGCGCCAAAAGAGAGA-3'; reverse: 5'-CTGGTTTTCCTTCAGCTGGAAGTGGT-3') and (forward: 5'-TGAAGGTCGGAGTCAACGGATTTGGT-3'; reverse: 5'-CATGTGGGCCATGAGGTCCACCAC-3') were used to amplify the VE-cadherin sequence and GAPDH [22]. PCR amplification was performed using the T1 Thermocycler (Biometra, Goettingen, Germany) as follows: 1 cycle of 94°C for 1 min; 25-30 cycles of 94°C for 1 min, 68°C for 2.5 min, and 72°C for 1 min; and 1 cycle of 72°C for 5 min [22]. Electrophoresis of the PCR product was performed on a 2% agarose gel containing 0.5 μg/ml ethidium bromides using 6 μl of the reaction. Results were normalized by the ratio of band density of specific product to GAPDH. The RT-PCRs were performed three times with independently derived samples.

### Western blot analysis

At the end of Genistein treatment, cells were rinsed twice with ice-cold PBS and then lysed with ice-cold lysis buffer (50 mM of Tris-HCl, pH7.5, 150 mM of NaCl, 0.5% NP-40, 1 mM of EDTA, 0.2 mM of PMSF, 100 μl/ml of proteinase inhibitor Aprotinin) for 30 min. The cell lysates were centrifuged at 12,000 g for 10 min at 4°C, and the supernatant was collected and stored at -80°C until use. Protein concentration was confirmed by using the Bradford assay. Equal amounts of protein were mixed with SDS sample buffer (0.125 M Tris-HCl, pH 6.8, 10% glycerol, 2% β-mercaptoethanol, 2% SDS and 0.1% bromophenol blue) and boiled for 5 min. Then samples were electrophoresed on SDS-PAGE and transferred to PVDF membrane using a standard protocol. The membrane was blocked in 5% non-fat dried milk for 2 h, rinsed and then incubated with antibody to human VE-cadherin (R&D Systems) 1 h at 37°C and overnight at 4°C. Excess antibody was then removed by washing the membranes in TBST (TBS containing 0.01% Tween 20) and membranes were incubated 1 h at 37°C with HRP-conjugated secondary antibodies. After being washed in TBST, bands were visualized by an enhanced chemiluminescence (ECL, Amersham Pharmacia Biotech) system and exposed to radiography film. Molecular weight was determined by comparison with molecular weight markers.

### Statistics

Statistical analyses were performed using software from SPSS for Windows 13.0 (SPSS Inc., Chicago, IL, USA). All data were described as mean ± SEM. To analyze the data statistically, we performed Student's *t*-test with analysis. Differences were considered significant when P < 0.05.

## Results

### VM structure in C918 cells and OCM-1A cells

**Figure 1:**
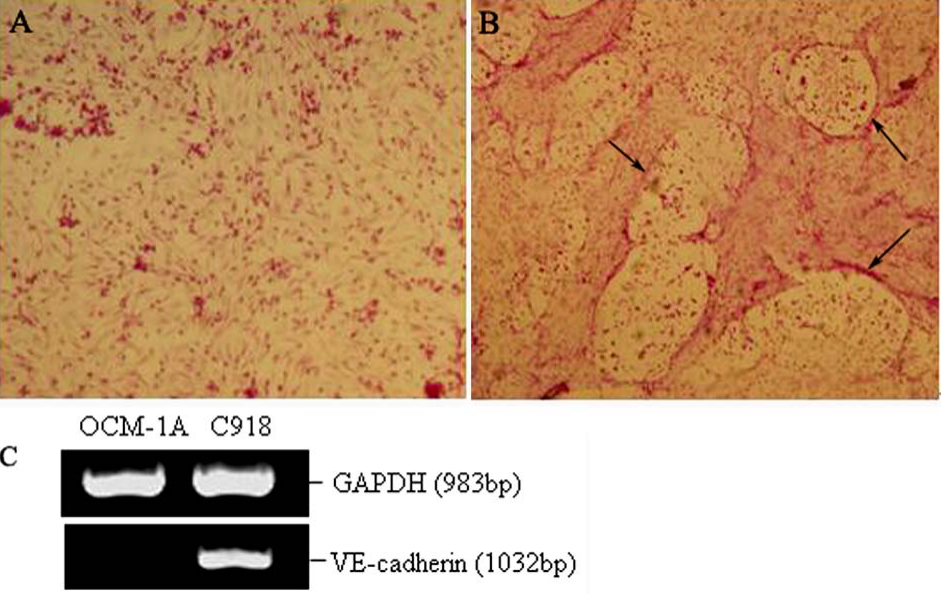
**Comparison of VM channels and the VE-cadherin mRNA level between C918 cells and OCM-1A cells**. (A) OCM-1A cells cultured in three-dimensional type I collagen gels do not form the VM network structure. (B) C918 cells have the ability to form the VM. (C) VE-cadherin was expressed by C918 cells but not OCM-1A cells. (A and B, Magnification: × 200)

As showed in Figure 1, the VM structure was found in highly aggressive uveal melanoma C918 cells cultured in three-dimensional type I collagen gels but not in poorly aggressive uveal melanoma OCM-1A cells. Moreover, C918 cells expressed the VE-cadherin and the contrary result appeared in OCM-1A cells.

### Effect of Genistein on the human uveal melanoma C918 cells growth

**Figure 2:**
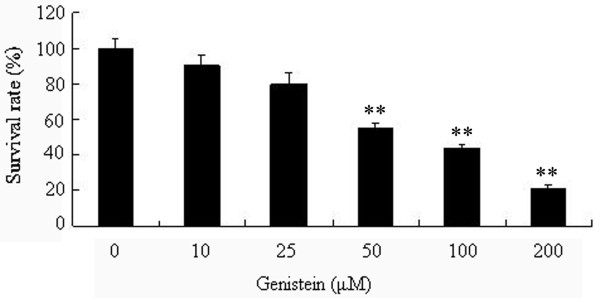
**Effect of Genistein on of human uveal melanoma C918 cells growth**. Proliferative activity of C918 cells was determined by the MTT assay after incubation for 48 h with Genistein (0-200 μM). **P < 0.01 vs. control.

After treatment for up to 48 h with various concentrations of Genistein (10, 25, 50, 100, 200 μM), the result displayed Genistein significantly decreased the cell survival in a dose-dependent manner (Figure 2). The cell morphology was observed under a phase contrast microscope following treatment with Genistein. Genistein significantly induced the spindle-cell morphology in C918 cells. At the final concentrations of 100 and 200 μM, Genistein leaded to 56.3 and 78.4% reductions in number of C918 cells, respectively. The control group was set at 100%.

### Evaluation of VM channel formation after Genistein treatment in vitro

**Figure 3:**
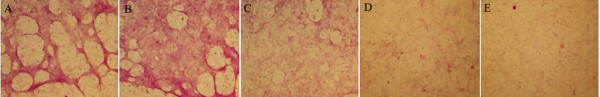
**The effect of Genistein on the vasculogenic mimicry of human uveal melanoma C918 cells on 3-D collagen I cultures**. PAS-stained images of C918 cells cultured on three-dimensional collagen I for 48 h in medium with different concentrations of Genistein. (A) control; (B) 25 μM Genistein; (C) 50 μM Genistein; (D) 100 μM Genistein; (E) 200 μM Genistein. At treatment groups with 25 μM and 50 μM concentrations of Genistein, C918 cells formed fewer VM matrix-association channels than do control. However, the groups treated with higher concentrations of Genistein (100 and 200 μM) did not form the VM channels. (Magnification: × 200)

After 48 h exposure to different concentrations Genistein, the ability of C918 melanoma cells to form VM channels was investigated using PAS staining (Figure 3). At the 25 μM and 50 μM of Genistein treatment groups, C918 cells formed fewer VM matrix-association channels than control. However, the groups treated with higher concentrations of Genistein (100 and 200 μM) did not form the VM channels.

### The regulation of microcirculation patterns by Genistein in vivo

**Figure 4:**
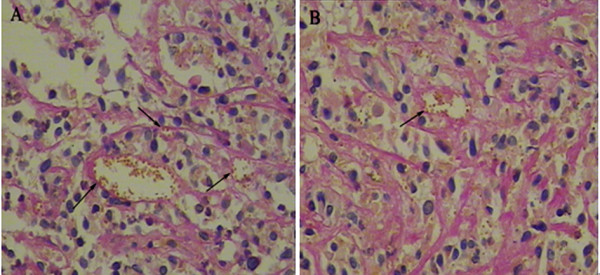
**CD34 and PAS double staining on the C918 human uveal melanoma xenograft sections**. (A) Control; (B) 75 mg/kg/day Genistein group; VM channel (arrow) is lined by PAS-positive materials and there are red cells in the center of the channels. (Magnification: × 400)

In order to further investigate the role of Genistein on VM formation of human uveal melanoma, we established ectopic model of human uveal melanoma in athymic nude mice. The result showed Genistein significantly inhibited the growth of xenograft in vivo. The inhibition rate of tumor growth for 75 mg/kg/day Genistein was 27.5% compared with the control group. VM in tumor tissue sections was evaluated (Figure 4) VM channels in C918-derived xenografts were significantly reduced in Genistein group compared with the control (P < 0.05) (Table 1).

**Table 1:** Comparison VM channels of xenograft specimens in the Genistein and control groups

Group*	**VM^# ^density** **(means ± S.E.M)**	***P***
Genistein (n = 5)	0.67 ± 0.17	*P*<0.05
Control (n = 5)	1.5 ± 0.23	

*Genistein group, Genistein was administered intraperitoneally (75 mg/kg/day) for 30 days. Control group received equivalent DMSO. Tumors were conducted immunohistochemical (CD34) and PAS double-staining to distinguish between endothelium-dependent microvessel and vasculogenic mimicry.

^# ^vasculogenic mimicry density.

### The influence of Genistein on the mRNA expression of VE-cadherin

**Figure 5:**
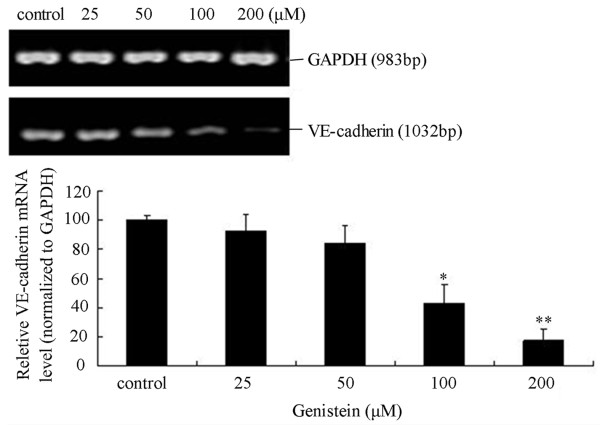
**Effect of Genistein on C918 cells VE-cadherin mRNA expression**. (A) The expression of VE-cadherin mRNA in C918 cells was examined by RT-PCR at 48 h after different concentration Genistein pretreatment (0, 25, 50, 100, 200 μM). (B) The results of VE-cadherin mRNA were expressed after normalized by β-actin. Data represent means ± S.E.M from three separate experiments. *P < 0.05, **P < 0.01 vs. control.

Semiquantitative RT-PCR was used to examine the VE-cadherin mRNA expression in C918 cells with different concentrations of Genistein. As demonstrated in Figure 5, VE-cadherin levels were significantly decreased in 100 and 200 μM Genistein-treated groups (P < 0.05 and P < 0.01, respectively). However, the 25 and 50 μM Genistein-treated groups slightly down regulated the VE-cadherin levels and no had statistics significance.

### The influence of Genistein on the protein expression of VE-cadherin

**Figure 6:**
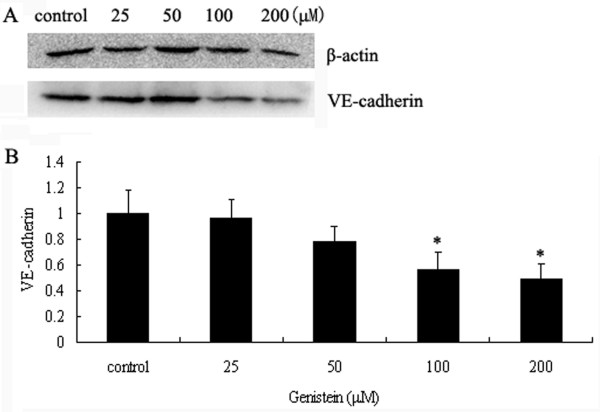
**Effect of Genistein on C918 cells VE-cadherin protein expression**. (A) The expression of VE-cadherin protein in C918 cells was examined by western blot at 48 h after different concentration Genistein pretreatment (0, 25, 50, 100, 200 μM). (B) The results of VE-cadherin protein were expressed after normalized by β-actin. The values were means ± S.E.M. n = 3. *P < 0.05 vs. control.

The VE-cadherin protein expression was assayed in C918 cells treated with different concentrations of Genistein (Figure 6). We found that 100 and 200 μM concentrations of Genistein could significantly inhibit VE-cadherin protein expression (P < 0.05). The levels were decreased to 55.9% ± 13.9% and 49.2% ± 11.2%, respectively, of that untreated with Genistein. However, the 25 and 50 μM Genistein slightly decreased the VE-cadherin protein (P > 0.05).

## Discussion

As a new tumor microcirculation pattern, VM differs from classically described endothelium-dependent angiogenesis. It is formed by aggressive melanoma tumor cells. Therefore, the VM channels maybe an additional target to treat solid tumors [3,25]. It has been demonstrated that several drugs could inhibit VM [22,26-28]. In this study, we found that Genistein could inhibit VM formation of uveal melanoma cells in vivo and in vitro.

Genistein has strong anticancer activities, including the inhibition of cell proliferation and angiogenesis, the induction of differentiation and apoptosis [29]. Numerous studies have reported the inhibitory effect of Genistein toward different tumor types. Moreover, Genistein was shown to inhibit growth of B16 mice melanoma cell in vivo and in vitro [30,31]. Our results suggested that Genistein inhibited the growth of C918 highly aggressive uveal melanoma cells in dose-dependent manner. The average quantity of VM in xenografts sections were significantly reduced in Genistein treatment group compared with the control. These results indicated that Genistein may have effect on VM formation of human uveal melanoma.

Further analysis suggested that one possible molecular mechanism of Genistein inhibited VM formation was related to down-regulation of VE-cadherin. Hendrix et al. found the expression of VE-cadherin by highly aggressive melanoma tumor cells leads to their ability to mimic endothelial cells and form VM in three-dimensional culture [20]. They thought VE-cadherin plays a critical role in the formation of VM by melanoma [20]. Hess et al. indicated VE-cadherin was involved in the initial signaling and regulation of the VM process. In present study, we indicated that the expression of VE-cadherin of C918 cells was lower in the Genistein treatment groups than the control group. In accordance with our results, previous studies also proved that Genistein was capable of reducing the expression of VE-cadherin [32,33]. High concentrations of Genistein (100, 200 μM) significantly reduced the expression of VE-cadherin and completely inhibited the formation of VM. Accordingly, Hendrix et al. also found no networks were formed when VE-cadherin expression was down-regulated [20]. In addition, recent study also suggested VM could be regulated through influencing the endothelium and epithelium-specific genes expression including VE-cadherin [34]. Consequently, we supposed the effect of Genistein on the formation of human uveal melanoma VM was mediated, at least partially, through reduction of VE-cadherin expression.

In addition, Genistein has been reported to inhibit angiogenesis in vivo and in vitro. Physiological connections between tumor cell VM and angiogenesis microcirculation have been demonstrated [35-39]. Thus, the decrease of angiogenesis may affect the VM channels.

## Conclusion

This study shows that Genistein could effectively inhibit the VM formation of C918 human uveal melanoma in vivo and in vitro. One of the mechanisms that Genistein inhibits VM is associated with down regulation of VE-cadherin. Our present study may provide preliminary evidence for future and wider research. Therefore, substantially more studies are needed to define the actions of Genistein on VM and find the effective therapeutic strategies of uveal melanoma and other cancers related to VM.

## Competing interests

The authors declare that they have no competing interests.

## Authors' contributions

RC carried out cell culture experiments, western blot analysis, RT-PCR and drafted the manuscript. QS performed the animal experiments and statistical analysis. LY participated in designing the study and revised the manuscript. HG contributed to image treatment and manuscript revision. YZ participated in manuscript revision. BW conceived of the study, participated in its design and coordination. All authors read and approved the final manuscript.
